# Elastofibroma Dorsi, a Rare Condition, with Challenging Diagnosis. Case Report and Literature Review

**DOI:** 10.3390/medicina57040370

**Published:** 2021-04-12

**Authors:** Octavian Neagoe, Cosmin Ioan Faur, Mihaela Ionică, Flavia Baderca, Roxana Folescu, Daniela Gurgus, Carmen Lăcrămioara Zamfir, Andrei Motoc, Mirela Loredana Grigoraș, Octavian Mazilu

**Affiliations:** 1 First Department of Surgery, Second Discipline of Surgical Semiology, “Victor Babeș” University of Medicine and Pharmacy, Eftimie Murgu Sq. Nr.2, 300041 Timișoara, Romania; dr.octavian.neagoe@gmail.com (O.N.); smtm.chirurgieoncologica@gmail.com (O.M.); 2 Department of Orthopedics, “Victor Babeș” University of Medicine and Pharmacy, Eftimie Murgu Sq. Nr.2, 300041 Timișoara, Romania; 3 Department of Functional Sciences, Discipline of Pathophysiology, “Victor Babeş” University of Medicine and Pharmacy, 300041 Timişoara, Romania; dr.mihaela.ionica@gmail.com; 4 Department of Microscopic Morphology, “Victor Babeș” University of Medicine and Pharmacy, Eftimie Murgu Sq. Nr.2, 300041 Timișoara, Romania; flaviabaderca@yahoo.com; 5 Department of Balneology, Medical Recovery and Rheumatology, “Victor Babeș” University of Medicine and Pharmacy, Eftimie Murgu Sq. Nr.2, 300041 Timișoara, Romania; folescu.roxana@umft.ro (R.F.); gurgus.daniela@umft.ro (D.G.); 6 Department of Morpho-Functional Sciences I, “Grigore T. Popa” University of Medicine and Pharmacy, 16 Universitatii Str., 700115 Iași, Romania; 7 Department of Anatomy and Embryology, “Victor Babeș” University of Medicine and Pharmacy, Eftimie Murgu Sq. Nr.2, 300041 Timisoara, Romania; amotoc@umft.ro (A.M.); grigoras.mirela@umft.ro (M.L.G.)

**Keywords:** benign tumor, lipoma, elastofibroma, elastic fibers, orcein

## Abstract

Elastofibroma dorsi (ED) is known as a particular clinical and biological entity. We report a case of a bilateral elastofibroma dorsi (ED) in a 65-year-old female who presented to the Department of General and Oncologic Surgery of Emergency Clinical Municipal Hospital Timisoara, Romania. The patient was symptomatic on the right side, presenting pain in the interscapulothoracic region associated with a variable tumoral mass, dependent on the position of the right arm. Imaging studies revealed a well-defined, bilateral tumoral mass with alternation of the muscular and fatty tissue. The initial diagnosis of lipoma was taken into consideration based on the CT scan and clinical findings. Surgical excision of the right subscapular tumor was performed without any postoperative complications. Microscopic examination of hematoxylin and eosin, Masson’s trichrome, and orcein stained slides revealed the diagnosis of ED. Considering the high rate of reported postoperative complications and the asymptomatic presentation of the contralateral subscapular mass, the patient underwent clinical and imagistic monitoring for the contralateral tumor. Due to its rare nature, ED is a difficult preoperative diagnosis that can, however, be suggested by its specific location and may require an accurate histopathological examination for a final diagnosis.

## 1. Introduction

Elastofibroma dorsi (ED) is known as a particular clinical and biological entity. ED usually develops between the inferior angle of the scapula and the thoracic wall. It is considered to appear secondary to mechanical lesions due to scapulothoracic biomechanics during abduction, being suggested at the same time to occur as a result of tissue aging [1]. Immunologic, biomechanical (33–100% are bilateral), and genetic factors (32% have familial history) are mentioned as predisposing causes for this condition [2,3]. The slow progression and benign character of the tumor, together with the variable clinical signs, the non-specific imaging findings, and the low incidence rate, make the histopathological exam crucial for an accurate diagnosis of ED. There is always a risk of misdiagnosing ED with other benign or malignant lipomatous (e.g., lipoma, chondroma, low-grade liposarcoma) or fibrous (e.g., desmoid fibromatosis, nuchal fibroma, and fibrolipoma) soft tissue tumors with possible localization in the periscapular area. [4,5].

Due to its rare appearance, the optimal therapeutical algorithm for ED is not standardized at this moment. A conservative approach is usually attempted for all the asymptomatic cases, marginal excision with proper management of the dead spaces and postoperative immobilization is required for symptomatic patients, taking care all the time to exclude the suspicion of soft tissue malignancy. The high rate of postoperative complications (seroma, hematoma) is seen mainly due to the localization of the lesion and the impossibility of complete immobilization of the excisional site [6,7].

## 2. Case Presentation

A 65-year-old female patient presented with persistent right paravertebral pain. The symptoms started 3–4 months before presentation, and there were no correlations with other associated comorbidities or preexistent treatments. A palpable tumoral mass appeared after 2 months at the level of the inferior pole of the scapula with dimensions that were dependable on the position and movement of the right thoracic limb. Among the preexistent comorbidities we mention: Cardio-vascular disease (hypertension, cardiac failure—NYHA II, chronic ischemic disease, history of ischemic stroke), psychiatric pathology (bipolar disorder), orthopedic conditions (bilateral coxarthrosis), and neurosurgical pathologies (bilateral sciatica with chronic pain in the lumbar region). The preexistent treatment included NSAID’s (nonsteroidal anti-inflammatory drug) and myorelaxant drugs for pain control of all orthopedic conditions together with the cardiac medication. There were no changes in evolution regarding the general or local clinical findings during follow-up, regardless of posture or therapeutic choice.

Clinically, at the right subscapular level, a firm, relatively well-defined tumoral mass could be palpated, with deep, submuscular localization, measuring approximately 7–8 cm in diameter, variable with the position of the right arm (increasing in size with adduction and internal rotation, respectively, with forward flexion of the shoulder). The amount of pain increased with the previously described movements, with partial functional impairment of the right shoulder.

Soft tissue ultrasound revealed the presence of a hyperechoic tumoral mass, relatively well-defined in the observable area, with weak vascularization at color Doppler sequence, protruding under the scapular plane and under the latissimus dorsi.

**Figure 1:**
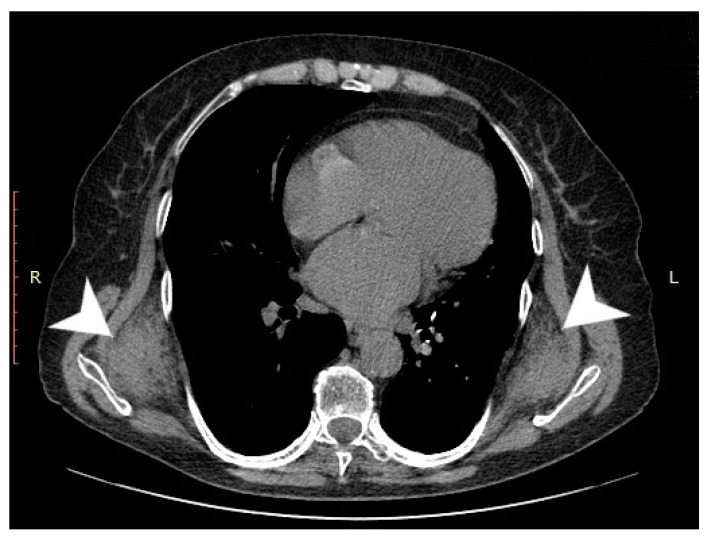
Axial CT view of the thoracic region at the level of the inferior third of the scapula. The tumoral masses are indicated by white arrows).

**Figure 2:**
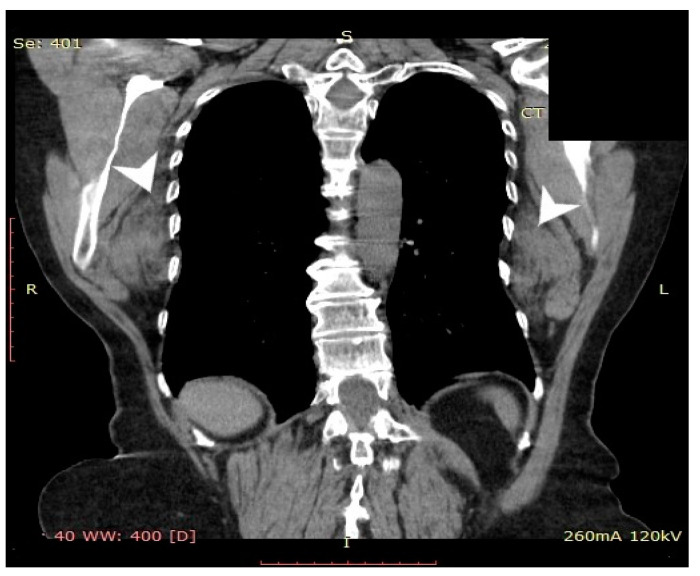
Coronal CT view of the thoracic region. The tumoral lesions are indicated by white arrows.

**Figure 3:**
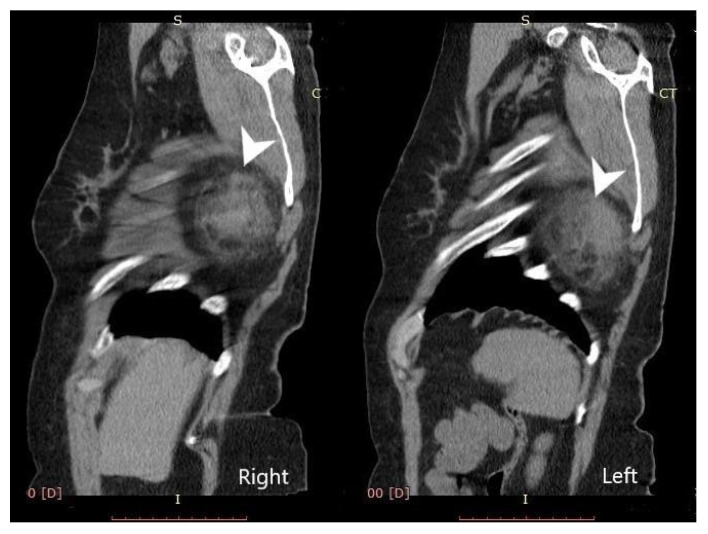
Bilateral sagittal view—elastofibroma dorsi (tumors are indicated by the white arrows).

Computed tomography (General Electric Brightspeed 16, Municipal Clinical Emergency Hospital Timisoara, Romania) revealed a bilateral tumoral mass with muscular and fatty density alternation, posterior to the serratus muscles and anterior to the latissimus dorsi muscles and the scapular plane (Figure 1, Figure 2 and Figure 3).

According to the clinical and imaging findings, the diagnosis of subscapular lipoma was suspected, and surgical treatment consisting of marginal excision of the tumor was proposed.

After proper preoperative evaluation and preparation, the patient was placed in left lateral decubitus with forward flexion of the arm. A parallel incision with the inferior margin of the scapula was performed. Following the dissection of the latissimus dorsi muscle fibers, a tumoral mass of 9/8/6 cm was found with the following macroscopic aspect: Polylobate surface, of firm consistency with rough areas, partially encapsulated (excepting the area of insertion at the level of the anterior scapular periosteum), with brownish color and yellowish, lipoma-like areas and white areas that comprised the whole tumoral mass, 6/6/5 cm in size, with a single vascular supply emerging from an artery located in the subscapular region. Intraoperative macroscopic characteristics of the tumor directed the previous clinical and imagistic diagnosis of lipoma towards a low-grade soft tissue sarcoma, and an extemporaneous microscopic exam of the tumoral tissue was performed.

The extemporaneous Papanicolau-stained smears examination did not reveal cellular or nuclear atypia, thus following surgical excision, drainage of the remaining cavity and closure of the anatomic planes was performed.

**Figure 4:**
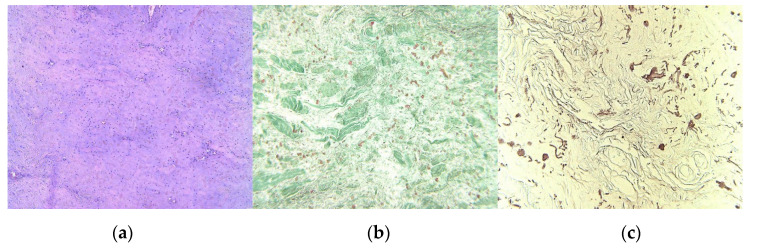
(**a**) Admixture of eosinophilic collagen fibers and birefractive thick, coarse, and sometimes globular distributed elastic fibers, H&E stain, original magnification (OM) × 10; (**b**) Acidophilic green different sized bundles of collagen fibers and a smaller quantity of red elastic fibers, Masson’s trichrome stain, OM × 10; (**c**) Red-brownish elastic fibers, with different sizes and aspects, orcein staining, OM × 10. (Nikon SMZ25 with Nikon’s Perfect Zoom System).

The tumoral tissue sample that was harvested during the surgical procedure was fixed in 10% neutral formalin solution and was included in histological paraffin, according to the histopathological protocol. There were performed 4-μm serial sections stained with Hematoxylin–Eosin (H&E); also, Masson’s trichrome and orcein stains were obtained. Microscopic H&E exam revealed a benign tumoral proliferation, with pale eosinophilic thick bands of collagen fibers, admixed with coarse, thick or globular groups, medium-size fusiform cells, with fibrillar eosinophilic cytoplasm, oval nuclei, some of them with irregular contour, others with sharp heads, fine chromatin pattern, equally distributed; small hyperemic blood vessels were also observed (Figure 4a). Masson’s trichrome staining colored collagen fibers in green, highlighting in red the elastic fibers (Figure 4b); the elastic fibers were better identified in red on orcein stained slides (Figure 4c). The histopathologic aspect suggests the diagnosis of ED.

There were no postoperative complications, drainage was maintained for 72 h, without the occurrence of postresection seroma or hematoma, and no signs of local inflammation. The postoperative functional score at 3 months was good according to OSS (Oxford Shoulder score) [8].

## 3. Discussion

ED is known to represent 2% of all primary tumors localized on the chest wall in adults [4,9], particularly in women. However, some imaging studies reported the presence of ED in 2% of asymptomatic adults over 60 years [9], respectively, below this percentage in case of symptomatic cases, with an incidence of 0.08% being reported in the database of the Royal Orthopedic Hospital in Birmingham (UK) and 1% recorded in the Japanese Soft Tissue Tumor Registry [10,11]. The extremely rare character of this pathology allows for the physician to suspect a profound lipoma rather than an ED, both having a similar progression from the clinical point of view [12,13].

These lesions are characterized by an asymptomatic evolution and tend to grow slowly in more than 50% of cases [7,14,15]. ED can be symptomatic too, with specific signs such as: Shoulder swelling, apparent increasing in size with shoulder adduction and lateral translation of the scapula, and pain [5,16]. In 60% of cases, subscapular elastofibromas develop on the right side, and in 66% of cases, this type of tumor are located bilaterally [2,17]. In the case of the development of a bilateral ED, the lesions can grow asynchronously.

The diagnosis cannot be sustained by clinical and anamnestic findings, the only possible indicator of an ED (compared to other tumoral conditions in the same region) being the symmetrical disposition of the periscapular lesions. In general, from the clinical point of view, in most cases, the tumoral mass is discovered randomly by the patient. In 10% of cases, the mass produces interscapulothoracic pain [2], as in this case report. The clinical examination can reveal a firm painless tumoral mass at the subscapular level, often bilateral, with partial limitation of the functionality of the shoulder, especially in abduction.

Soft tissue ultrasound, as well as CT or magnetic resonance imaging (MRI) do not reveal specific elements that would suggest the diagnosis of ED, except the location, the symmetrical aspect of the lesions, and, respectively, the density pattern. Positron emission tomography-computed tomography (PET-CT) with fluorodeoxyglucose (18F-FDG) examination has shown its utility in identifying ED lesions, in case of patients undergoing screening studies for cancer, with a reported frequency of 1.66% [18].

A fine-needle or core biopsy can be done under local anesthesia confirming the lesion benignity, procedure recommended by several authors [2,19]. However, due to the inhomogeneous character of ED (CD34 mesenchymal cells that can evolve to endothelial cells, myocytes or adipose lines [20]), needle biopsy should be performed under ultrasound guidance for a significant increase in accuracy of the harvesting procedure in order to be able to analyze a relevant sample of tumoral tissue. It is rather the collaboration between clinicians and radiologists that can suggest the preoperative suspicion of ED [21,22]. Several papers in the recent literature indicate that imaging studies should be enough if the lesion is unilateral or asymmetrical, painful, with hard consistency, and rapidly progressing in volume [23,24,25]. Development of a tumoral mass in that specific region can include a number of differential diagnosis such as: Hemangioma, neurofibroma, malignant histiocytoma, lipoma [7,26,27], and requires an MRI with gadolinium contrast, thus the malignant tumors can be differentiated [23,24,25].

In these conditions, the therapeutical algorithm varies between surgical excision for symptomatic patients and surveillance for asymptomatic cases [10]. In general, if the ED is asymptomatic, the therapy is resumed to simple observation, but if the lesion is large (>5 cm) surgical marginal resection can be considered as the optimal treatment. The previously described patient fits both situations, as the protrusive, highly painful lesion was surgically removed, while the contralateral asymptomatic lesion remains in clinical and imagistic monitoring [6,7,28]. Due to the high risk of local seroma occurring in the postoperative period (35.9–87.5%), as a consequence of regional biomechanics and increased dead spaces resulted from the excision of those large tumors, surgical treatment is addressed strictly to symptomatic cases, as well as to those with potential malignant progression [12,13,29]. There have been no cases of malignant proliferation following ED described in the literature [6], but there have been reported several ED cases of local recurrence after incomplete surgical removal of the entire tumoral tissue [9,29].

There is still a debate even these days whether ED’s are authentic neoplasms or pseudotumors developing from an abnormal soft tissue [2,19], seen most often in manual workers because of the repetitive trauma (playing the major role in the etiology of these tumors). However, there have been described cases of the same tumor with other localizations. Genetic causes together with inherent enzymatic defects or systemic involvement are considered the main factors that are involved in the development of multiple elastofibromas. [27].

## 4. Conclusions

This article presents a rare case of bilateral elastofibroma dorsi with different asymmetrical comparative evolution of both sides. The asymptomatic variant with no influence on the scapulothoracic motion can be observed on one side in contrast with the symptomatic type with significant influence on the shoulder mobility on the other side.

ED is a rare tumoral entity that can be diagnosed based on the clinical and imagistic findings, especially through its specific location, but possibly requiring histochemical staining for a final diagnosis.

Surgical marginal resection should be reserved only for symptomatic cases with significant discomfort for the patient during daily activities because of the increased rate of local complications. Although related problems, residual seroma or hematoma in the post excisional space and the prolonged postoperative rehabilitation interval following this latissimus-split procedure are among the most commonly reported complications.

## References

[1] Giebel G.D., Bierhoff E., Vogel J. (1996). Elastofibroma and pre-elastofibroma—A biopsy and autopsy study. Eur. J. Surg. Oncol..

[2] Nagamine N., Nohara Y., Ito E. (1982). Elastofibroma in Okinawa. A clinicopathologic study of 170 cases. Cancer.

[3] Naylor M.F., Nascimento A.G., Sherrick A.D., McLeod R.A. (1996). Elastofibroma dorsi: Radiologic findings in 12 patients. AJR Am. J. Roentgenol..

[4] Freixinet J., Rodriguez P., Hussein M., Sanroman B., Herrero J., Gil R. (2008). Elastofibroma of the thoracic wall. Interact. Cardiovasc. Thorac. Surg..

[5] Muratori F., Esposito M., Rosa F., Liuzza F., Magarelli N., Rossi B., Folath H.M., Pacelli F., Maccauro G. (2008). Elastofibroma dorsi: 8 case reports and a literature review. J. Orthop. Traumatol..

[6] Muramatsu K., Ihara K., Hashimoto T., Seto S., Taguchi T. (2007). Elastofibroma dorsi: Diagnosis and treatment. J. Shoulder Elbow Surg..

[7] Daigeler A., Vogt P.M., Busch K., Pennekamp W., Weyhe D., Lehnhardt M., Steinstraesser L., Steinau H.U., Kuhnen C. (2007). Elastofibroma dorsi—differential diagnosis in chest wall tumours. World J. Surg. Oncol..

[8] Haragus H., Prejbeanu R., Patrascu J., Faur C., Roman M., Melinte R., Timar B., Codorean I., Stetson W., Marra G. (2018). Cross-cultural adaptation and validation of the Romanian Oxford Shoulder Score. Med. Baltim..

[9] Brandser E.A., Goree J.C., El-Khoury G.Y. (1998). Elastofibroma dorsi: Prevalence in an elderly patient population as revealed by CT. AJR Am. J. Roentgenol..

[10] Chandrasekar C.R., Grimer R.J., Carter S.R., Tillman R.M., Abudu A., Davies A.M., Sumathi V.P. (2008). Elastofibroma dorsi: An uncommon benign pseudotumour. Sarcoma.

[11] Nagano S., Yokouchi M., Setoyama T., Sasaki H., Shimada H., Kawamura I., Ishidou Y., Setoguchi T., Komiya S. (2014). Elastofibroma dorsi: Surgical indications and complications of a rare soft tissue tumor. Mol. Clin. Oncol..

[12] Parratt M.T., Donaldson J.R., Flanagan A.M., Saifuddin A., Pollock R.C., Skinner J.A., Cannon S.R., Briggs T.W. (2010). Elastofibroma dorsi: Management, outcome and review of the literature. J. Bone Joint Surg. Br..

[13] Mortman K.D., Hochheiser G.M., Giblin E.M., Manon-Matos Y., Frankel K.M. (2007). Elastofibroma dorsi: Clinicopathologic review of 6 cases. Ann. Thorac. Surg..

[14] Kastner M., Salai M., Fichman S., Heller S., Dudkiewicz I. (2009). Elastofibroma at the scapular region. Isr. Med. Assoc. J..

[15] Oueslati S., Douira-Khomsi W., Bouaziz M.C., Zaouia K. (2006). Elastofibroma dorsi: A report on 6 cases. Acta. Orthop. Belg..

[16] Nishio J., Isayama T., Iwasaki H., Naito M. (2012). Elastofibroma dorsi: Diagnostic and therapeutic algorithm. J. Shoulder Elbow Surg..

[17] Jarvi O., Saxen E. (1961). Elastofibroma dorse. Acta Pathol. Microbiol. Scand. Suppl..

[18] Blumenkrantz Y., Bruno G.L., Gonzalez C.J., Namias M., Osorio A.R., Parma P. (2011). Characterization of Elastofibroma Dorsi with (18)FDG PET/CT: A retrospective study. Rev. Esp. Med. Nucl..

[19] Enjoji M., Sumiyoshi K., Sueyoshi K. (1985). Elastofibromatous lesion of the stomach in a patient with elastofibroma dorsi. Am. J. Surg. Pathol..

[20] Hisaoka M., Hashimoto H. (2006). Elastofibroma: Clonal fibrous proliferation with predominant CD34-positive cells. Virchows Arch..

[21] Faccioli N., Foti G., Comai A., Cugini C., Guarise A., Mucelli R.P. (2009). MR imaging findings of elastofibroma dorsi in correlation with pathological features: Our experience. Radiol. Med..

[22] Go P.H., Meadows M.C., Deleon E.M., Chamberlain R.S. (2010). Elastofibroma dorsi: A soft tissue masquerade. Int. J. Shoulder Surg..

[23] Haykir R., Karakose S., Karabacakoglu A. (2007). Elastofibroma dorsi: Typical radiological features. Australas Radiol..

[24] Malghem J., Baudrez V., Lecouvet F., Lebon C., Maldague B., Vande Berg B. (2004). Imaging study findings in elastofibroma dorsi. Joint Bone Spine.

[25] Pop D.L., Nodiţi G., Abu-Awwad A., Maliţa D.C., Zamfir C.L., Grigoraş M.L., Vermeşan D., Prejbeanu R., Faur C.I., Hărăguş H.G. (2018). Alveolar rhabdomyosarcoma in an adolescent male patient—case report and current perspectives. Rom. J. Morphol. Embryol..

[26] Alouini R., Allani M., Harzallah L., Bahri M., Kraiem C., Tlili-Graies K. (2005). Elastofibroma: Imaging features]. J. Radiol..

[27] Le Goudeveze S., Chapuis O., Scherier S., Jancovici R. (2008). Elastofibroma dorsi: A differential diagnosis in subscapular soft tissue tumors. Presse Med..

[28] Fibla J., Molins L., Marco V., Perez J., Vidal G. (2007). Bilateral elastofibroma dorsi. Joint Bone Spine.

[29] Briccoli A., Casadei R., Di Renzo M., Favale L., Bacchini P., Bertoni F. (2000). Elastofibroma dorsi. Surg. Today.

